# Dry Heat and Conservative Cooking

**Published:** 1912-06-01

**Authors:** 


### INSTITUTIONAL HOUSEKEEPING.

### Dry Heat and Conservative Cooking.

The object of this comparatively modern system of preparing food is to preserve entire the juices and salts of the food, some of which are of so delicate and elusive a nature that they defy analysis. When food is subjected to heat in the open air as in roasting before a fire, or in an atmosphere constantly renewed as in baking, or plunged in water as in boiling, these valuable ingredients may to a large extent either evaporate or be washed out. Conservative cookery is the process of preparing food not in boiling water, nor in steam arising from boiling water, but by dry heat. The apparatus needed is a double pan or double cooker. The simplest of these are constructed on the principle of the ordinary water-bath, of which the French *bain-marie* is an example. They consist of a double pan, one inside the other, the outer one being filled with water which is kept at boiling-point, while the inner vessel contains the food. The heat only penetrates slowly to the latter, which never reaches a temperature of 212°, and hence there is no risk of the food being dried up or burned. There are special utensils on the market for this style of cooking, one of which we brought to the notice of our readers a short time back. The double saucepan in ordinary use for boiling milk can be made to answer the purpose for small quantities, provided the escape of steam from the outer pan is controlled. Should the inner one not fit closely the lid can be inverted and a weight set on it, but the steam must not be retained without any possibility of escape or there will be risk of an explosion. The pressure should be relieved at intervals when the inner pan is examined to ascertain if more boiling water is required. Other pots in use are those of the Warren pattern, or the Wellbank's boilerettes, in which the pressure of the steam is safely regulated.

In large institutions the double boilers can be utilised for conservative cookery, care being taken that no more liquid is added to the food than is to be actually served and consumed with it, otherwise the valuable juices and extractives will be diluted or lost. Where, however, these double boilers are surrounded by steam some amount of liquid must be present in the inner pan or the metal will crack. Milk, porridge, stews with gravy, soup, etc., can be thus safely prepared. In pans with a water surround no liquid need be added at all, and the juices which are found when the cooking is done must be served in the dish, after due seasoning, and, if desired, a little thickening. It is a good plan, however, to add an ounce or two of butter, or milk, or stock, according to the nature of the food which is being prepared—vegetable, fish, or meat.

The process of cooking in this conservative manner is a good deal longer than by ordinary methods, when the food is brought into actual contact with boiling water or steam. But, on the other hand, there is a great saving of labour in a process which obviates the necessity for constant stirring and watching. Most kinds of foods improve in tenderness by slow cooking and hence become easier of digestion. The gain in nutritive value is remarkable. For instance, the water in raw vegetables varies from 78 per cent. to 90 per cent., and this fact conduces to their low nutritive value compared with their bulk. They absorb still more water while being boiled in the ordinary way, and at the same time they are continuously drained of their valuable mineral constituents. If cooked slowly in dry heat the whole of their contents are preserved undiluted, and those who have never tasted them prepared scientifically are unaware of the fine flavourings which are destroyed by the ignorance of cooks.

Again, the flavouring ingredients of fish are even more easily dissolved out by water than those of meat, being far more delicate. And for this reason no satisfactory result can be expected from submerging and cooking fish in water. Prepared in a double cooker, fish is an ideal food for a convalescent, and hence, it goes without saying, for those in good health also, and the fact that the fish has not been boiled in the orthodox fashion will not be discovered save by its extra palatability.

The methods of conservative cookery have so much to recommend them in their simplicity and their economy, that it is much to be wished they were more commonly used in institutions for the sick and in large households where the aim should be to make use of every particle of nourishment present in the food.

### Housekeeping Queries.

Domestic Staff in Nurses' Home.

Sister Dorothy.—With thirty nurses in the Home you will require a cook, kitchenmaid, dining-room maid, and two housemaids. For dinner the housemaids will dress and help in waiting, and the duty of answering the door-bell will be divided. The housekeeper must make out a time-table right through the day for each member of the staff, and be constantly vigilant that it is strictly adhered to. An outworker or "scrubber" should be engaged once or twice a week if there is any wide extent of stone stairs and passages and for polishing floors. It will be found that this deranges the routine less than attempting to fit this kind of heavy work in with the regular duties of the staff.

Allowance of Tea.

"Niggard."—You must reckon that each ounce of tea will make six pints, and measure it out according to heads for each meal. The important matter in brewing tea for large numbers is to tie it in very loose muslin bags so that every leaf is exposed to the boiling water. The bag or bags should be moved about swiftly by a spoon in the receptacle used, and it is a good plan to examine the contents afterwards to see whether there is any dry tea in the centre, as that will show that loss has occurred. The bags must be well rinsed and hung up to dry, or a musty flavour will be imparted to subsequent brews.

### TO OUR READERS.

Hardly a week passes in institution life when some problem does not occur to worry the superintendent or one of her staff. Let us know whenever you are in difficulties, and give us the opportunity of suggesting a remedy. Inform us of any devices you find useful for saving labour, and make known for the benefit of others the triumphs over circumstances which you occasionally achieve.

